# Research on the Transformer Failure Diagnosis Method Based on Fluorescence Spectroscopy Analysis and SBOA Optimized BPNN

**DOI:** 10.3390/s25072296

**Published:** 2025-04-04

**Authors:** Xueqing Chen, Dacheng Li, Anjing Wang

**Affiliations:** 1 Institutes of Physical Science and Information Technology, Anhui University, Hefei 230601, China; q22301351@stu.ahu.edu.cn; 2 Anhui Institute of Optics and Fine Mechanics, Hefei Institutes of Physical Science, Chinese Academy of Sciences, Hefei 230031, China; dcli@aiofm.ac.cn; 3 Key Laboratory of General Optical Calibration and Characterization Technology, Hefei Institutes of Physical Science, Chinese Academy of Sciences, Hefei 230031, China

**Keywords:** transformer failure diagnosis, fluorescence spectroscopy, back propagation neural network (BPNN), secretary bird optimization algorithm (SBOA)

## Abstract

The representative dissolved gases analysis (DGA) method for transformer fault detection faces many shortcomings in early fault diagnosis, which restricts the application and development of fault detection technology in the field of transformers. In order to diagnose early failure in time, fluorescence analysis technology has recently been used for the research of transformer failure diagnosis, which makes up for the shortcomings of DGA. However, most of the existing fluorescence analyses of insulating oil studies combined with intelligent algorithms are a qualitative diagnosis of fault types; the quantitative fault diagnosis of the same oil sample has not been reported. In this study, a typical fault simulation experiment of the interval discharge of insulating oil was carried out with the new Xinjiang Karamay oil, and the fluorescence spectroscopy data of insulating oil under different discharge durations were collected. In order to eliminate the influence of noise factors on the spectral analysis and boost the accuracy of the diagnosis, a variety of spectral preprocessing algorithms, such as Savitzky–Golay (SG), moving median, moving mean, gaussian, locally weighted linear regression smoothing (Lowess), locally weighted quadratic regression smoothing (Loess), and robust (RLowess) and (Rloess), are used to smooth denoise the collected spectral data. Then, the dimensionality reduction techniques of principal component analysis (PCA), kernel principal component analysis (KPCA), and multi-dimensional scale (MDS) are used for further processing. Based on various preprocessed and dimensionally reduced data, transformer failure diagnosis models based on the particle swarm optimization algorithm (PSO) and the secretary bird optimization algorithm (SBOA) optimized BPNN are established to quantitatively analyze the state of insulating oil and predict the durations of transformer failure. By using the mathematical evaluation methods to comprehensively evaluate and compare the effects of various algorithm models, it was found that the Loess-MDS-SBOA-BP model has the best performance, with its determination coefficient (R^2^) increasing to 99.711%, the root mean square error (RMSE) being only 0.27144, and the other evaluation indicators also being optimal. The experimental results show that the failure diagnosis model finally proposed in this paper can perform an accurate diagnosis of the failure time; the predicted time is closest to the true value, which lays a foundation for the further development of the field of transformer failure diagnosis.

## 1. Introduction

The transformer is an important infrastructure for transmission and distribution in a power system, monitoring its operation status, strengthening the ability to detect faults, especially in the early stage of faults, the timely detection of faults, and early warnings, which is conducive to allowing maintenance personnel to quickly take countermeasures, minimize losses, and keep the power system operating stably [1,2]. Insulating oil is an insulating material stored in the oil tank of an oil-immersed transformer, and when the transformer is at fault, the state of the insulating oil changes, and based on this mechanism, the dissolved gas analysis method (DGA) has been widely used to study transformer failure, and the faults can be diagnosed according to the gas change in the oil [3,4].

However, in early fault warnings, DGA is constrained by the following:

1. The characteristic gas content does not change significantly, does not exceed the gas standard warning value, and cannot issue an early warning for latent hidden dangers in time;
2. The intelligence is low and the auxiliary discrimination method is not flexible;
3. The processing process is complex and the detection cycle time is long, which makes it impossible to quickly perform an early fault diagnosis [5,6,7].

The research on this technology needs to be gradually improved or replaced by other more efficient new technology methods, such as the latest research that has proposed a new method for the detection of dissolved gases and the use of fiber-enhanced Raman spectroscopy technology to detect a variety of decomposition gases in environmentally friendly gas-insulated power equipment, so as to perform the operation status evaluation of gas-insulated power equipment [8].

Facing the shortcomings of gas analysis methods in identifying the early faults of oil-immersed transformers, fluorescence technology has been applied to the field of transformer failure detection in some studies, due to its advantages of the large amount of information, fast detection speed, high sensitivity, and that fact that it does not need to be in direct contact with oil samples. Studies have shown that the generation of and change in fluorescence signals in transformer oil are related to the change in the concentration of aromatic hydrocarbons, which are fluorescent substances [9,10]. When some electrical and thermal failures arise within transformers, the aromatic substances in the oil crack and the spectral intensity weakens, so the fluorescence analysis method of insulating oil can realize the timely monitoring of transformer oil deterioration [11,12,13,14]. At present, most of the reported research on the failure detection of power transformers is qualitative diagnosis. Pengcheng Yan, Rongying Dai, and Feng H et al. used laser-induced fluorescence (LIF), built a LIF system to collect the insulating oil spectra of different transformer fault types, and fused a variety of intelligent algorithms to establish a classification model to classify and identify the common fault types of power transformers (electrical faults, thermal faults, local moisture faults, lightning faults, water inlet damage faults, and short circuit faults) [15,16,17,18].

Due to the different causes of faults, the positions and intensities of oil fluorescence spectral characteristic peaks of different fault types are often very different and can even be effectively identified by the eye alone, so they can often achieve a good fault classification effect [19,20,21]. However, there are few fault quantitative diagnostic studies on transformer oil of the same type of fault with no obvious spectral difference, which cannot accurately predict the fault time and evaluate the severity of the fault type. For the quantitative detection of faults, the fluorescence double-color ratio method proposed by Zhao Yue et al. and the ultra-high-sensitivity fault detection method proposed by Mu L et al. on this basis are relatively new methods that have been reported so far. However, they all rely on fluorescence bi-characteristic peak information, which requires the spectra to exhibit the characteristics of doublets, and this approach may not be suitable for complex fluorescence spectra with single or multiple peaks [22,23].

In order to solve the problem of the quantitative diagnosis of transformer early fault duration time, this paper proposes a universally applicable and accurate quantitative diagnosis method of transformer faults by combining fluorescence spectral characteristic analysis and the artificial intelligence algorithm.

## 2. Materials and Methods

**Figure 1:**
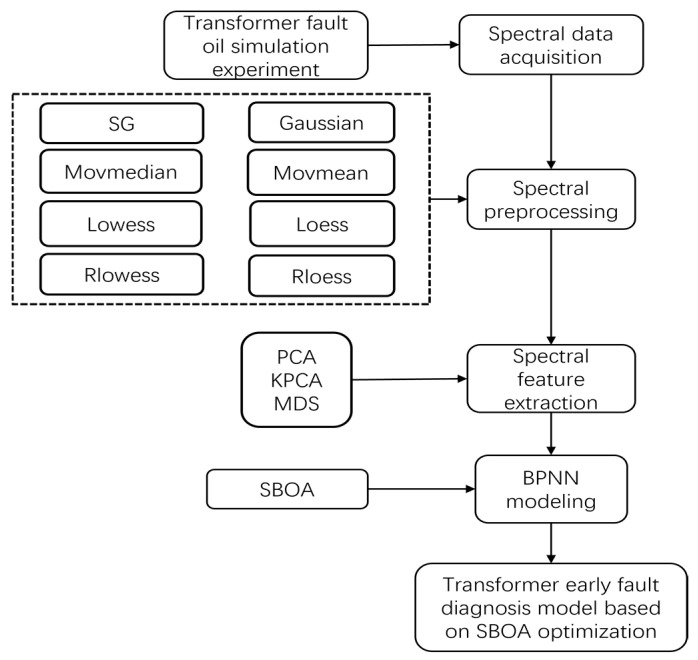
Technical flow chart.

The technical route is shown in Figure 1. Firstly, the interval discharge experiment was simulated. After the early fault oil samples were obtained, the fluorescence spectral data under different fault durations were collected, and several preprocessing algorithms were used for smooth denoising: Savitzky–Golay (SG), moving median, moving mean, gaussian filtering, locally weighted linear regression smoothing (Lowess), locally weighted quadratic regression smoothing (Loess), and robust (RLowess) and (Rloess) signal smoothing algorithms. Then, redundant information was removed and the data dimension was reduced by principal component analysis (PCA), kernel principal component analysis (KPCA) [24], and multi-dimensional scale (MDS) dimensionality reduction algorithms. Based on the preprocessed data, an error back propagation neural network (BPNN) model was established, then the network’s weight and threshold parameters were optimized by using the secretary bird intelligence optimization algorithm (SBOA). Compare the diagnostic results of different models to verify the effectiveness of the transformer failure diagnosis model based on the SBOA optimized BPNN proposed in this study and perform the quantitative diagnosis of the early failure time of transformers.

### 2.1. Materials

#### 2.1.1. Experimental Samples

The new Karamay oil was filtered and discharged to simulate the typical fault of the interval discharge, and the oil samples of early failure were obtained and stored in a sealed container. A total of 6 oil samples with different fault degrees were obtained, and we collected 10 pieces of spectral data for each faulty oil sample. All faulty oil samples used were prepared by experiments at the Institute of Electrical Engineering, Chinese Academy of Sciences. The storage and spectral acquisition were carried out in a dark room to prevent the influence of light. The faulty oil information is shown in Table 1.

#### 2.1.2. Spectral Acquisition

The fluorescence spectroscopy of each faulty oil sample was collected, and the detection instrument was the OmniFluo 900 fluorescence spectrometer produced by Zolix Instrument Co., Ltd (Beijing, China). A 75 W continuous xenon lamp was used as the excitation light source. The collection parameters are shown in Table 2.

#### 2.1.3. Original Spectral Analysis

**Figure 2:**
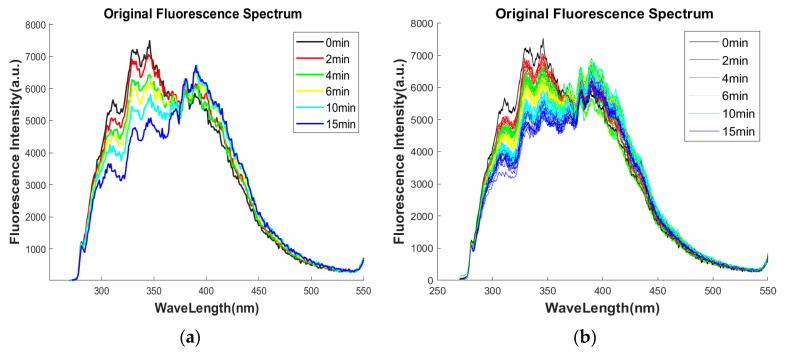
Original spectral analysis: (**a**) fluorescence spectra at different fault times; (**b**) all original fluorescence spectral data.

As shown in Figure 2a, the original fluorescence spectra at different degrees of failure were acquired at a fixed excitation wavelength of 280 nm, and a random piece of spectral data was selected at each failure time to plot. The fluorescence characteristic bands of the emission spectrum are mainly in the range of 270 nm–550 nm; the difference in fluorescence intensity is mainly in the range of 300 nm–450 nm. With the increase in the discharge time, the intensity of fluorescence gradually decreased [25]. The typical characteristic peaks of the new oil spectrum are as follows: characteristic peak-1: EM is 311 nm, fluorescence intensity is 5658 a.u., characteristic peak-2: EM is 329 nm, fluorescence intensity is 7167 a.u., characteristic peak-3: EM is 346 nm, fluorescence intensity is 7509 a.u., characteristic peak-4: EM is 390 nm, and fluorescence intensity is 5861 a.u. After the wavelength of 420 nm, the spectrum gradually converges, and no other characteristic peaks appear. The intensity values of characteristic peaks 1, 2, and 3 gradually reduced with the increase in the discharge time, and the variation pattern of the fluorescence intensity of characteristic peak 4 is not obvious. The reason for this phenomenon is that the insulating oil of the power transformer is mainly composed of hydrocarbons, such as alkanes and cycloalkanes, and in the case of arc and local overheating, there is a phenomenon of carbon–hydrogen bond breakage or carbon–carbon bond breakage, and the broken carbon atoms and hydrogen atoms are recombined through complex chemical reactions to generate other chemical substances that do not have fluorescent properties, and the aromatic hydrocarbon concentration decreases, resulting in the fluorescence intensity of the insulating oil decreasing with the deepening of the degree of failure.

All original spectral data collected by the spectrometer are shown in Figure 2b. The pyrolysis degree of aromatic hydrocarbons of different faulty transformer oils with shorter fault interval times is relatively similar, the peak position and fluorescence intensity of the fluorescence spectral curves overlap, and the degree of overlap between some curves is relatively high. The spectral signal and the aromatic hydrocarbon concentration changes may have a complex nonlinear relationship. In order to correlate the spectral information with the degree of failure and directly detect the degree of failure of unknown oil samples with the information acquired from the known data, the BPNN with a good nonlinear modeling ability was used for the regression analysis.

### 2.2. Methods

#### 2.2.1. Spectral Preprocessing

Because spectral data acquisition may be performed in a relatively complex environment, the spectrum will inevitably experience interference from factors, such as the noise of high-power instruments, the noise of laser-device heat dissipation, and the noise of the external environment, resulting in the spectrum containing useless interference information, which is superimposed on the spectral image as unsmooth curves with obvious burrs, which affects the data quality and the recognition effect of the subsequent model. Therefore, it is essential to preprocess the original signal to enhance the spectral quality and filter out influences of the characteristic factors of the non-faulty oil itself. In this study, Savitzky–Golay (SG) smoothing, moving median filtering (Movmedian), moving mean filtering (Movmean), Gaussian filtering, locally weighted linear regression smoothing (Lowess), locally weighted quadratic regression smoothing (Loess), and robust (RLowess) and (Rloess) signal smoothing algorithms were used to effectively filter out high-frequency noise and improve spectral smoothness and the signal-to-noise ratio. The SG filtering algorithm uses the least squares polynomial to fit the signal within the filter window, and then uses the fitting function to calculate the smoothed data points, resulting in smooth denoising. The Movmedian, Movmean, and Gaussian filter calculate the median, moving average, and Gaussian-weighted average, respectively, for the data within the window. Lowess and Loess are algorithms that combine weighted linear least squares with first-order and second-order polynomials, respectively, to fit the polynomial regression curves in the intervals. Rlowess and Rloess are more robust forms and filter better for outliers that deviate farther.

#### 2.2.2. Dimensionality Reduction

The full-band fluorescence spectral data of the sample have many features and contain a large amount of redundant information, which will greatly increase the computational complexity and parameter tuning difficulty, which is not conducive to the rapid and accurate output prediction results [26]. Moreover, there are obvious differences in the importance of different wavelengths for spectral analysis, and non-critical spectral band data affect the model and cannot achieve a good prediction effect. Considering the actual requirements for the speed and accuracy of transformer fault diagnosis, this study uses several dimensionality reduction algorithms for various preprocessed data: principal component analysis (PCA), kernel principal component analysis (KPCA) based on the kernel function, and the multi-dimensional scale (MDS) analysis dimensionality reduction method to simplify the data while retaining the important features of the data, which is helpful to enhance the training speed and performance of the model.

#### 2.2.3. BP Neural Network

The BPNN is a type of multi-layer feedforward neural network trained by backpropagating errors, and has strong nonlinear mapping capabilities [27]. In the training stage, the input samples are transformed nonlinearly to generate an output, and the output error is calculated. Then, the error is backpropagated to continuously adjust the parameters of the network, and the error is gradually reduced through repeated training until the network achieves the desired output results. The structure is divided into an input layer, hidden layer, and output layer, and the neurons are connected by weight and thresholds. The specific number of layers and neurons in each layer can be set arbitrarily. Hidden layer neurons can map the main attributes of the input sample into the output, and the number of neuronal nodes has a crucial impact on the final performance of the model.

In order to select the optimal number of hidden layer neurons and build the optimal network structure, this study used the dimensionality reduction data as the input and the cumulative failure time as the output, and built a network with different numbers of hidden layer neurons to compare the training effect. According to Equation (1), m and n are the number of neurons in the input layer and output layer, respectively, and t is taken as the tuning constant between the integers in the range of 1–10.

(1) Hidden Num=sqrt(m+n)+t,

The optimal number of hidden layer nodes is determined to be in the range of 3–13, and the MSE of the training set corresponding to the number of neurons under each independent experiment is recorded.

**Figure 3:**
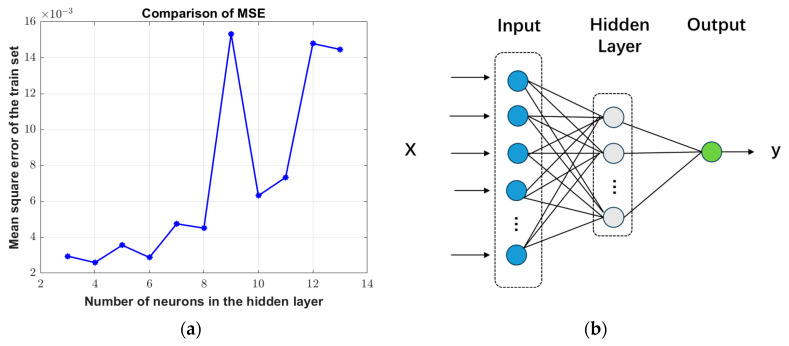
(**a**) MSE comparison under different numbers of hidden layer neurons; (**b**) BP neural network structure.

As shown in Figure 3a, comparing the magnitude of the MSE of the training set corresponding to the different number of nodes in the hidden layers, the optimal number of nodes is 4, and the training effect of the model is the best. The MSE is only 0.0025891, so the BP neural network model with the corresponding structure is constructed as shown in Figure 3b.

#### 2.2.4. PSO Optimization

In the training process, the BPNN gradually looks for the optimal parameters, such as the connection weight and threshold, through error backpropagation, but it is likely to fall into the local optimum, which will affect the final effect of the network. Moreover, the initialized network parameters are random and the initial value has a great impact on the network performance. Therefore, this study uses the intelligent optimization algorithm to optimize the weight and threshold parameters of the network model, so as to obtain a better model failure prediction effect.

Particle swarm optimization (PSO) is a classical global optimization algorithm based on swarm intelligence, which is often used for various parameter optimization work. The algorithm simulates the predatory behavior of a population of birds and finds the optimal solution for the search space through the information sharing and cooperation between particles in the population [28].

The motion of the particles is guided by the individual optimal solution (pBest) and the global optimal solution (gBest), and their position and velocity are updated according to Equations (2) and (3):

(2) $$v_{i}^{t+1}={wv}_{i}^{t}+c_{1}r_{1}\left({pBest}_{i}-x_{i}^{t}\right)+c_{2}r_{2}(gBest-x_{i}^{t})$$

(3) $$x_{i}^{t+1}=x_{i}^{t}+v_{i}^{t+1}$$

where $v_{i}^{t}$ and $x_{i}^{t}$ represent the velocity and position of particle i in generation t, w is the inertia weight, $c_{1}$ and $c_{2}$ are learning factors, ${pBest}_{i}$ represents the optimal position of particle i in the historical iteration, and gBest is the best position of the whole population.

The flow of the algorithm is as follows:

Step 1: Initialize the position and velocity of the particles randomly, and set the inertia weight, acceleration constant, maximum number of iterations, and other parameters of the algorithm.

Step 2: Calculate the fitness value for each particle based on the objective function.

Step 3: Update the individual optimal solution ${pBest}_{i}$ and the global optimal solution gBest using the velocity and position update formulas.

Step 4: Start the cycle from step 2 until the maximum number of iterations or the error meets the requirements, and the result is output.

**Figure 4:**
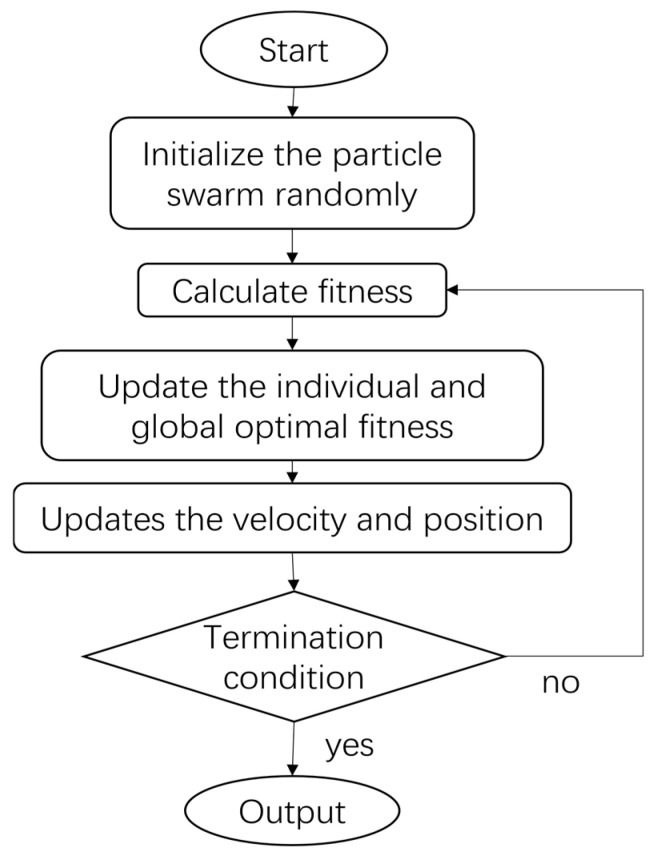
Flow chart for PSO algorithm.

The flow chart is shown in Figure 4:

#### 2.2.5. SBOA Optimization

The secretary bird optimization algorithm (SBOA) is a novel swarm bionic intelligent optimization algorithm, which is separated into two stages, the exploration stage and the development stage, which is conceived from the survival and hunting method of the secretary bird to hunt prey and escape from pursuit, with a fast convergence speed and good stability [29]. The flow of the SBOA algorithm is shown in Equations (4)–(11).

Step 1: Initialization stage:

Initialize the population, N; the dimension of the solution space, Dim; the maximum number of iterations, T; initialize the position, $X_{i,j}$ of the secretary bird randomly in the search space; and calculate the fitness function, Fi.

(4) $$X_{i,j}={lb}_{j}+r*({ub}_{j}-{lb}_{j}),$$

where ${ub}_{j}$ and ${lb}_{j}$ are the upper and lower bounds.

Step 2: Exploration stage P1:

1. Hunting for prey:

The differential evolution strategy is used to create new solutions, which boosts the diversity and global search ability of the algorithm and avoids falling into a local optimum.

(5) $$While\;t<\frac{1}{3}T,\;x_{i,j}^{new\;P1}=x_{i,j}+(x_{random\_1}-x_{random\_2})*R_{1},$$

2. Exhaust Prey:

Brownian Motion (RB) is adopted to simulate random behaviors of secretary birds circling around prey that exhausts their stamina. $x_{best}$ is the present optimal value.

(6) RB=randn(1,Dim),

(7) while 13T<t<23T,xi,jnew P1=xbest+exp((tT)^4)∗(RB−0.5)∗(xbest−xi,j)

3. Attack prey:

In order to enhance the global search ability, the weighted Levy flight strategy, RL, is introduced. The update method is as follows, where (1 − t)^(2 * t) is the nonlinear disturbance factor, which is used to achieve a balance between the exploration and development stages.

(8) While t>23T, xi,jnew P1=xbest+((1−tT)^(2∗tT))∗xi,j∗RL,

In this stage, the position of $X_{i}$ in the search space is updated as follows:

(9) $$X_{i}=\left\{\begin{array}{c} X_{i}^{new,P1}\;,\;\;if\;F_{i}^{new,P1}<F_{i}\\ X_{i}\;\;\;\;,\;\;\;\;\;\;\;\;\;\;\;\;\;\;\;\;\;\;\;\;\mathrm{else} \end{array}\right.,$$

Step 3: Development stage P2: there are two strategies for escaping from pursuit.

C1: hide in the environment in disguise;

C2: flee or fly away quickly.

(10) $$X_{i,j}^{new,P2}=\left\{\begin{array}{c} C1:x_{best}+\left(2*RB-1\right)*{\left(1-\frac{t}{T}\right)}^{2}*x_{i,j}\;\;\;\;\;,\;\;\;\;\;\;\;\;\;\;if\;rand<r_{i}\\ C2:x_{i,j}+R_{2}*(x_{random}-K*x_{i,j})\;\;\;\;\;\;\;\;\;\;\;\;,\;\;\;\;\;\;\;\;\;\;\;\;\;\;\;\;\;\;\;\;\;\;\;\mathrm{else} \end{array}\right.,$$

(11) $$X_{i}=\left\{\begin{array}{c} X_{i}^{new,P2}\;,\;\;if\;F_{i}^{new,P2}<F_{i}\\ X_{i}\;\;\;,\;\;\;\;\;\;\;\;\;\;\;\;\;\;\;\;\;\;\;\mathrm{else} \end{array}\right.,$$

Step 4: until the iterative termination condition is satisfied, the best solution $x_{best}\;$ is output.

**Figure 5:**
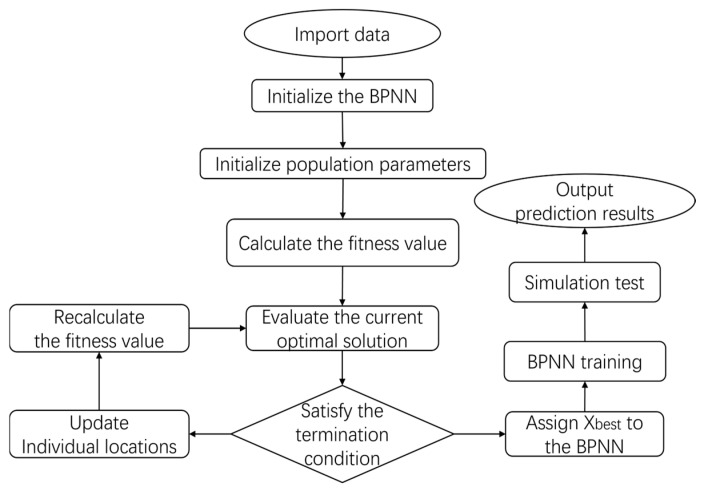
Flow chart for SBOA-BP failure diagnosis model.

In this study, the SBOA was used to treat the connection weights and thresholds between the layers of the neural network as the subjects in the population, and the global best solution found after iteration stop was assigned to the BPNN. Thus, the optimized SBOA-BP failure diagnosis model was obtained. The flow chart is shown in Figure 5.

## 3. Results and Discussion

### 3.1. Spectral Preprocessing

**Figure 6:**
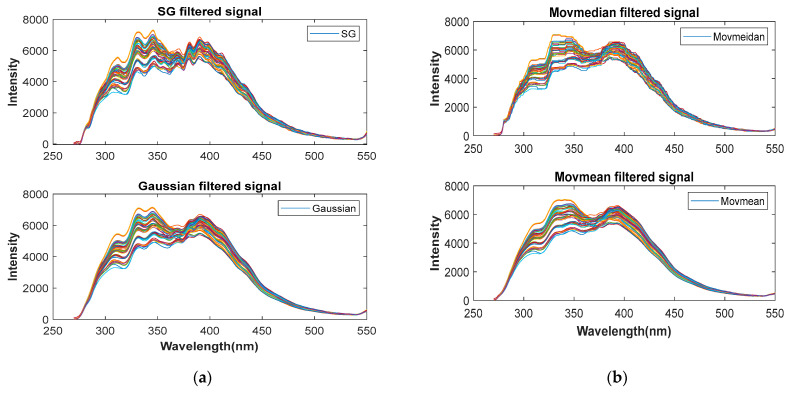

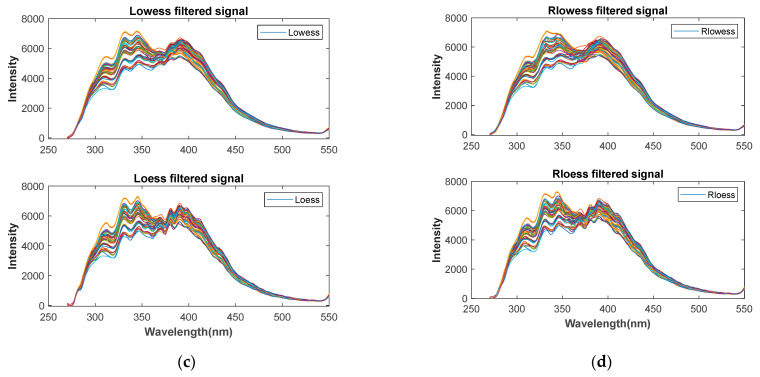
Preprocessed images. (**a**) SG and Gaussian filtered signals; (**b**) moving median and moving mean filtered signals; (**c**) Lowess and Loess filtered signals; (**d**) Rlowess and Rloess filtered signals.

Comparing several preprocessed images in Figure 6, it can be seen that, after SG, Gaussian, Lowess, Loess, and Rloess filtering, the noise of the middle and high frequencies in the signal is effectively suppressed, and the curve becomes smoother. The Movmedian filter calculates the median in each window, the signal is still not smooth enough after preprocessing, and there are still many interferences in the curve. And Movmean and Rlowess have higher smoothness after pretreatment. However, it is still difficult to select the best filtering method from the image alone. Each oil sample is randomly divided into a training set and a test set according to the proportion, a neural network model is established, and a suitable smooth denoising algorithm is selected according to the regression prediction ability of the model. In order to improve the credibility and avoid the overfitting of the model and affect the generalization ability, an independent test set is used to evaluate the actual performance of the model. In Table 3, the prediction results of the test set after the original spectrum and various preprocessed spectral modeling tests are recorded, among which the prediction value of the unprocessed Original-BPNN model deviates the most from the real value, and the prediction effect is the worst: the RMSE of test set is as high as 2.7423 and the model’s R^2^ is only 70.477%. However, compared with the Original-BP, the prediction effect of the model after various smooth denoising preprocessing tests improves. The best of these is Rlowess-BP, whose R^2^ increases to 96.294% and RMSE decreases to 0.97161; the worst is the Movmedian model, whose R^2^ also increases to 87.987% and the RMSE to 1.7493. However, due to the large spectral dimension, the overall prediction error of the model is still large. Considering the practical application, the requirements for the accuracy and precision of the transformer failure time analysis require spectral dimensionality reduction to improve the accuracy of the results.

### 3.2. Dimensionality Reduction

**Figure 7:**
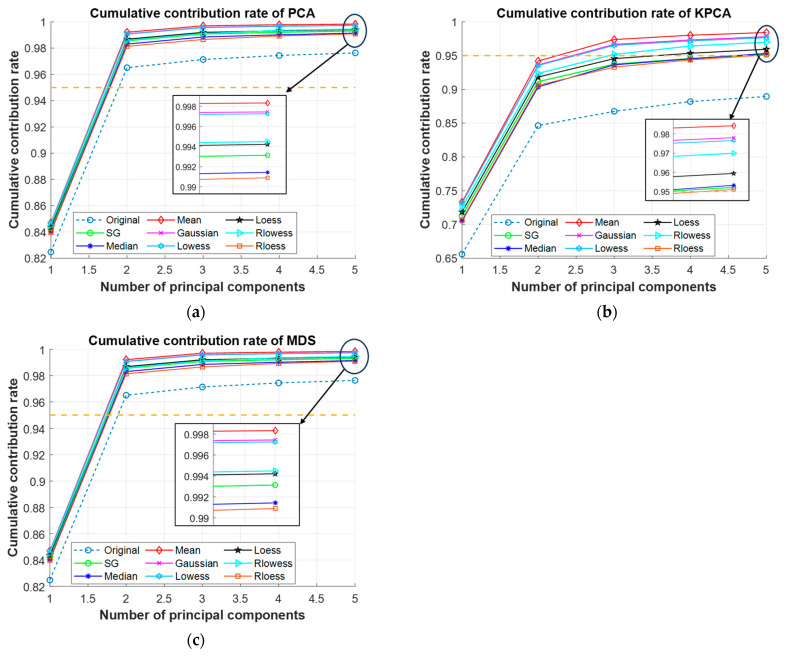
Cumulative contribution of principal components under PCA, KPCA, and MDS: (**a**) PCA; (**b**) KPCA; (**c**) MDS.

Figure 7 shows the number of principal components and the corresponding cumulative contributions of the data under various preprocessing steps. When there is one principal component, the cumulative contribution of all models does not reach 85%, and then the cumulative contribution rate gradually increases with the increase in the number of selected principal components. Taking KPCA dimensionality reduction as an example, when there is one principal component, the cumulative contribution rate of the Mean-KPCA model is the highest, reaching 73.3159%, and the other models are lower than this. The cumulative contribution rates of all preprocessed models are higher than those of the original data; the cumulative contribution rate of the Original-KPCA is the lowest, only 65.6076%. When the number of principal components increases to 5, the cumulative contribution gradually stabilizes, and the cumulative contribution of all data preprocessed models are higher than 95%. At this time, the cumulative contribution of Mean-KPCA is still the highest, which is 98.4293%; the lowest is Rloess-KPCA, which is 95.1081%; and the cumulative contribution rate of untreated Original-KPCA is only 88.9571%. So, the number of principal components is selected as 5 for subsequent modeling and identification.

**Figure 8:**
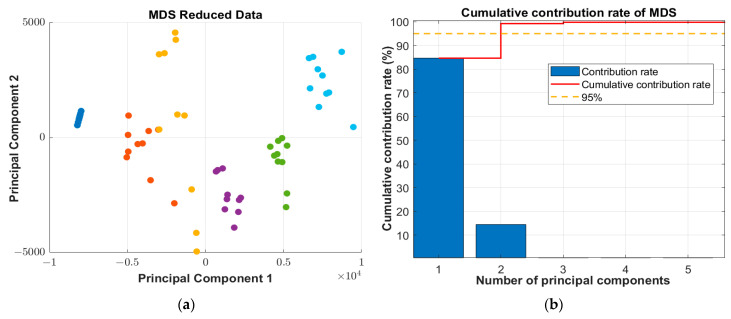
(**a**) First and second component scores after MDS dimensionality reduction; (**b**) cumulative contribution rate after MDS dimensionality reduction.

Comparing the cumulative contribution of different models, under the same dimensionality reduction method, the cumulative contribution of all preprocessed models improved compared with the original data without pretreatment. And in several dimension reduction methods, all the cumulative contributions after Movmean pretreated reached the highest value, and all the cumulative contributions after Rloess pretreatment were the lowest. In general, Movmean-MDS has the highest cumulative contribution rate among all models, with the first five principal components reaching 99.8327%, and the lowest is Original-KPCA, only 88.9571%. Figure 8 shows the cumulative contribution rate of Movmean-MDS and the data distribution of the first two principal components after dimensionality reduction.

### 3.3. BP Neural Network

Then, the BP neural network model is established to fit the transformer failure time, and the data visualization and model evaluation parameters are compared, so as to find the most suitable smooth preprocessing and feature extraction methods. The prediction results of each model test set are recorded in Table 4, and the one with the best effect is SG-PCA-BP with an R2 of 99.411% and RMSE of 0.38728 under the same PCA dimensionality reduction method. The worst model is the Rlowess-PCA-BP with an R^2^ value of 96.234% and RMSE of 0.97948. The Gaussian-KPCA-BP pretreatment model has the best effect under the same KPCA method, with an R^2^ of 97.799% and RMSE of 0.74868. The worst effect model is Lowess-KPCA-BP with an R^2^ of 88.559% and RMSE of 1.7071. The best model under MDS reduction is Rlowess-MDS-BP with an R^2^ of 98.633% and RMSE of 0.59011, while the worst is the SG-MDS-BP model with an R^2^ of 95.575% and RMSE of 1.0616. The model’s prediction value fluctuates up and down the real value, the overall error fluctuation is large, and the regression effect of the model needs to be enhanced.

### 3.4. PSO Optimization

**Figure 9:**
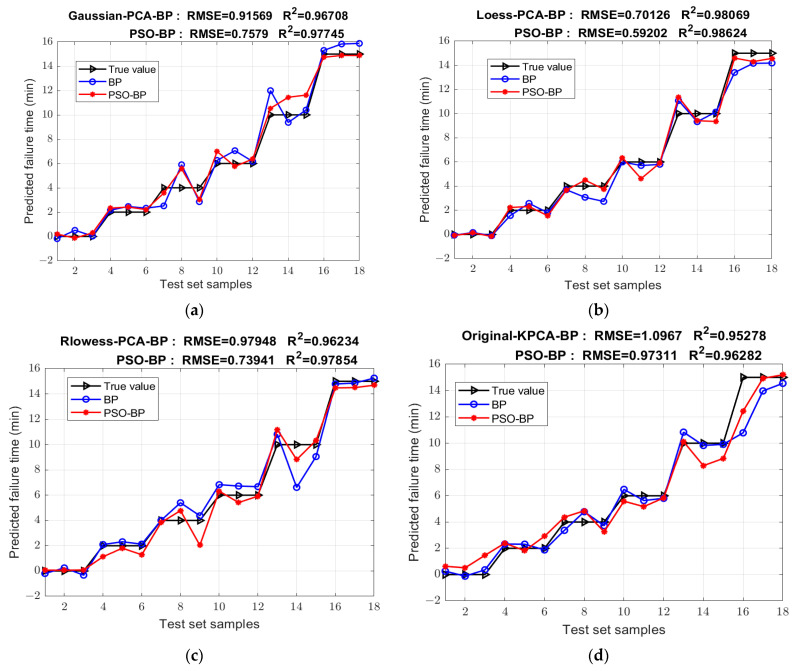

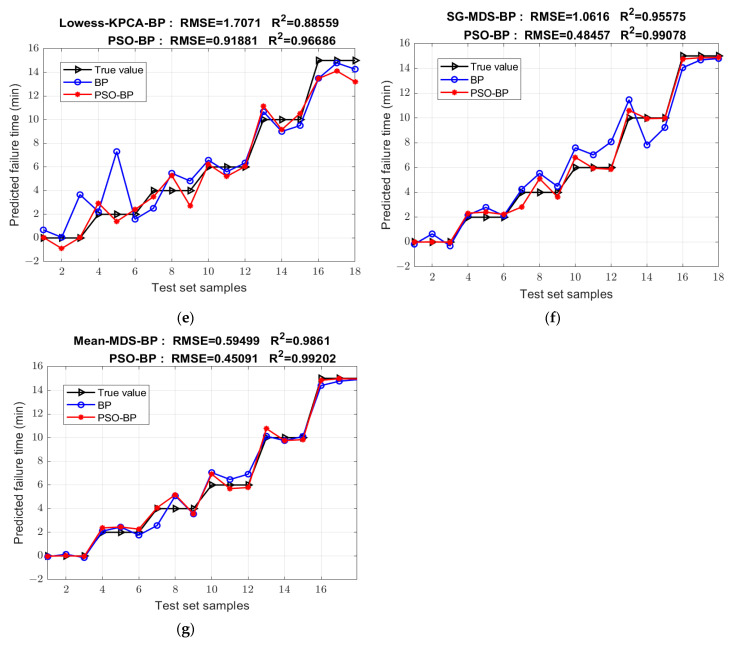
Comparison of models before and after PSO optimization under different preprocessing methods. (**a**) Gaussian-PCA; (**b**) Loess-PCA; (**c**) Rlowess-PCA; (**d**) Original-KPCA; (**e**) Lowess-KPCA; (**f**) SG-MDS; (**g**) Mean-MDS.

The transformer fault diagnosis model based on the PSO optimized BP neural network is established to quantitatively analyze the state of insulating oil. The prediction results of the following seven neural network models established under different preprocessing dimensionality reductions are improved compared to those before optimization, while the results of other models are not improved. The comparison of the prediction results before and after optimization is shown in Figure 9, and the performance metrics of each optimized model are shown in Table 5. Among them, the Mean-MDS-PSO-BP model has the best performance: R^2^ is 99.202% and RMSE is 0.45091. As a mainstream intelligent optimization algorithm, PSO has been widely used for various optimization work for many years, and its optimization of the model needs to be replaced by other optimization algorithms with better results.

### 3.5. SBOA Optimization

**Figure 10:**
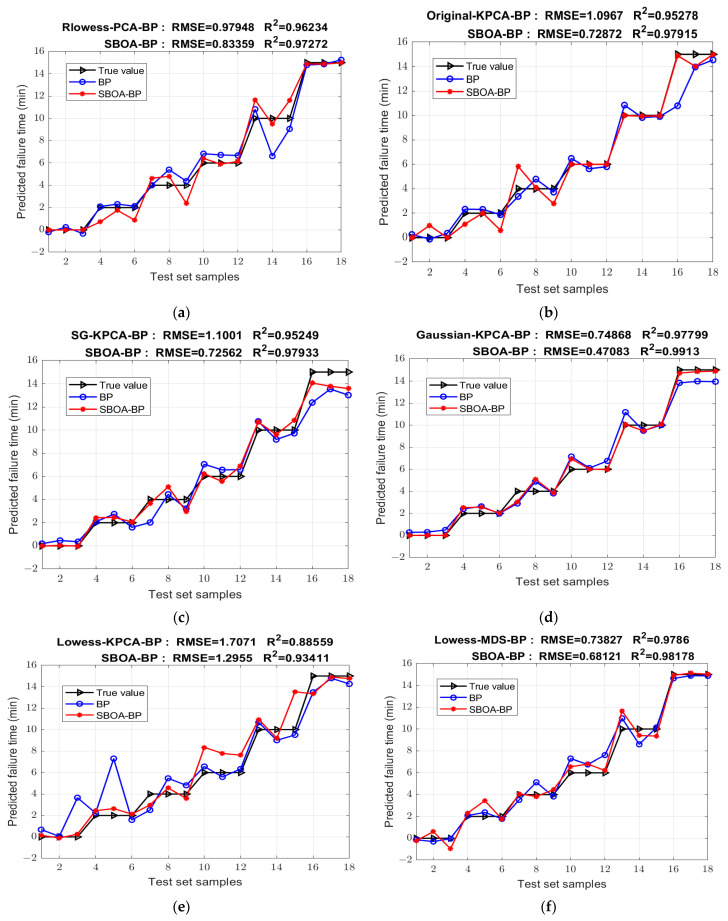

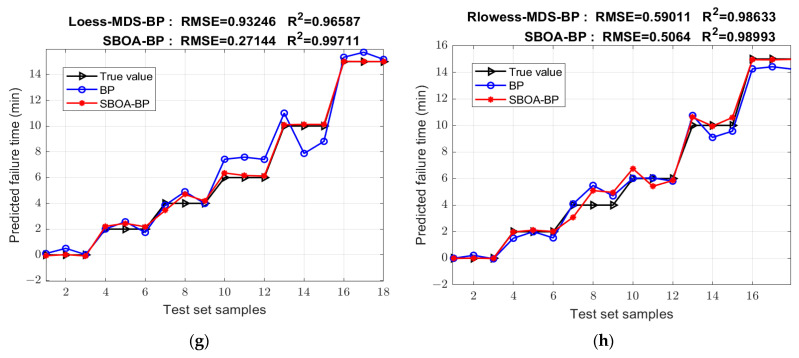
Comparison of models before and after SBOA optimization under different preprocessing methods. (**a**) Rlowess-PCA; (**b**) Original-KPCA; (**c**) SG-KPCA; (**d**) Gaussian-KPCA; (**e**) Low-ess-KPCA; (**f**) Lowess-MDS; (**g**) Loess-MDS; (**h**) Rlowess-MDS.

**Figure 11:**
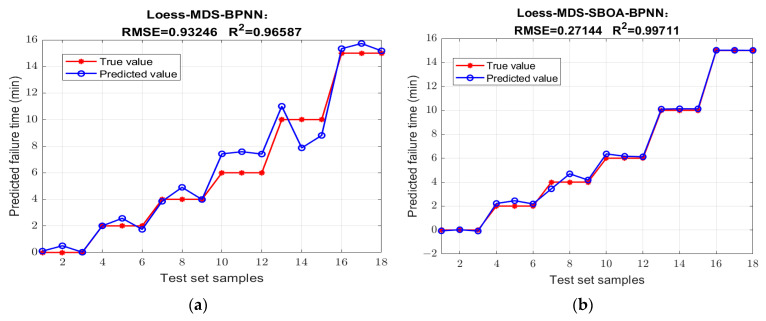
Comparison of the best Loess-MDS-SBOA-BP model before and after optimization. (**a**) Lo-ess-MDS-BPNN; (**b**) Loess-MDS-SBOA-BPNN.

The transformer failure detection model based on the SBOA optimized BPNN is established to quantitatively analyze the state of insulating oil. The prediction results of the following neural network models established under different preprocessing dimension reduction methods are improved greatly to varying degrees after SBOA optimization compared with those before optimization. The comparison of the prediction results before and after optimization is shown in Figure 10, and the performance indicators of each model are shown in the Table 6. Among them, the Loess-MDS-SBOA-BP model has the best performance: R^2^ is increased to 99.711%, the RMSE is reduced to 0.27144, and the other evaluation indexes are also the best, and the predicted failure time is the closest to the true value. Compared with the best Mean-MDS-PSO-BP model after POS optimization, this model has a higher determination coefficient and a better regression fitting effect, compared to the untreated Original-BP, whose R^2^ is only 70.477% and RMSE is 2.7423, which maximizes the prediction ability. The RMSE is reduced to 1/10, as shown in Figure 11.

**Figure 12:**
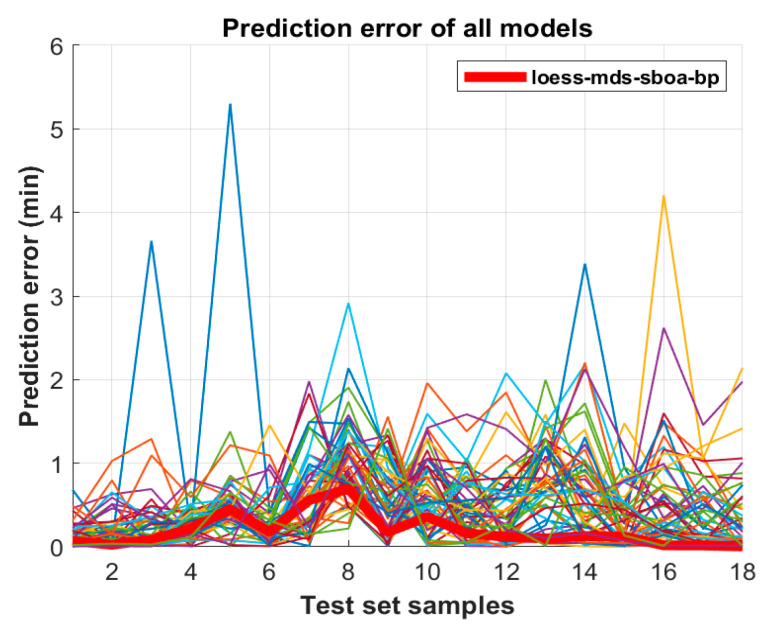
The prediction error curves of all models in the test set.

**Figure 13:**
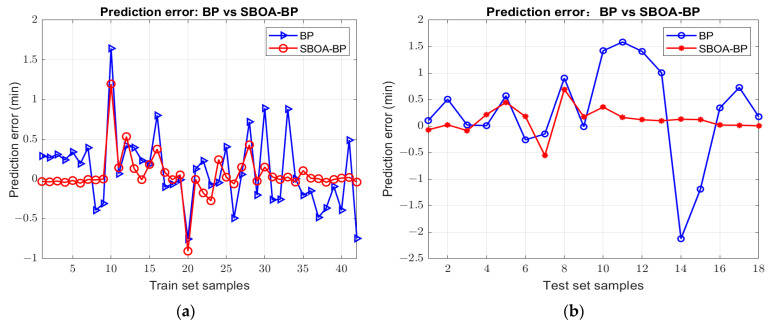
Prediction error of Loess-MDS-SBOA-BPNN before and after optimization. (**a**) Training set; (**b**) test set.

The prediction error curves of all models are shown in Figure 12. In general, the errors of samples 2–6 and 14–16 fluctuate greatly, while in the remainder of the samples, the errors deviate from the true value slightly. Compared with the BP fault diagnosis model, the error fluctuation between the predicted value and the real value of the optimized SBOA-BP failure detection model is small, and the regression prediction effect of the model is greatly improved. Among them, the Loess-MDS-SBOA-BP model still has the smallest fluctuation in the error curve; the error curves of the training set and the test set before and after optimization are shown in Figure 13. The experimental results show that the Loess-MDS-SBOA-BP model is the best model suitable for the quantitative diagnosis of early faults in the transformer, which can provide an accurate diagnosis of the fault time and provide a basis for the further development of the domain of transformer failure diagnosis.

### 3.6. Fluorescence Double-Color Ratio

**Figure 14:**
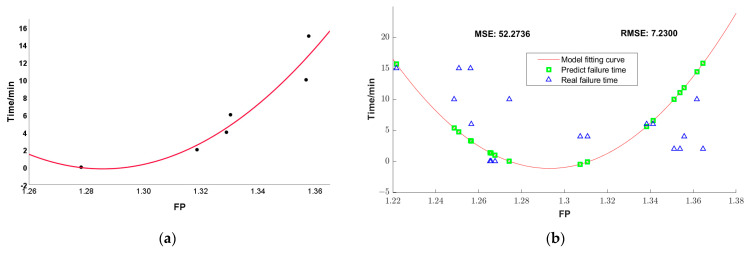
Double-color proportional failure diagnosis model of insulating oil. (**a**) Model fitting curve; (**b**) failure time diagnosis effect.

The fluorescence double-color ratio method is a method that has been proposed in recent years for quantitative fault diagnosis based on the analysis of fluorescence characteristic peak intensity data. Based on the analysis of the fluorescence signals in this experiment, two characteristic peaks in the ranges of 308–311 nm and 329 nm–332 nm are extracted as the double-color information extraction bands, and the feature proportions (FP) are shown in Table 7. The fluorescence two-color ratio fault diagnosis model established by least squares fitting is T = 2500.64*(FP)^2 − 6430.51*(FP) + 4113.90, the correlation coefficient is 0.9157, and the fitting curve is shown in Figure 14.

Using the established double-color ratio diagnostic model, the failure time of the oil sample is deduced according to the fluorescence ratio value calculated by the measurement for the same test set. The result is shown by the green square mark in the figure, and the blue triangle mark is the real fault time. The MSE of the prediction results of the double-color ratio model is 52.2736 and RMSE is 7.2300, and this model’s predicted values differ significantly from the true values. The reason is that the changes in the fluorescence multi-peak spectrum is complex, and the fluorescence ratio value cannot express the fault characteristics of the oil samples well. The result shows that the double-color ratio model is less effective in the application of multi-peak complex fluorescence spectra.

## 4. Conclusions

In this paper, fluorescence analysis and fault diagnosis are carried out for the interval discharge fault of transformer insulating oil, and the spectral data are directly correlated with the quantitative analysis results of the transformer fault. Comparing the diagnostic effects of the models under different preprocessing methods, the experimental results show that the Loess-MDS-SBOA-BP failure diagnosis model optimized by the SBAO algorithm has the best prediction effect, which can accurately predict the fault duration time. The R2 value of the model reaches 99.711% and the MSE of the model’s test set is only 0.073678. The proposed model enables maintenance personnel to find potential safety hazards in time at the early stage of a fault and carry out fault repairs, and also provides a research basis for more adequate transformer oil fluorescence data collection methods and further development in the field of transformer fault diagnosis in the future.

## Figures and Tables

**Table 1:** The information of the faulty transformer insulating oil samples.

Degree	Acquisition Time	Numbers
0 min	1 March 2024	10
2 min	1 March 2024	10
4 min	1 March 2024	10
6 min	1 March 2024	10
10 min	1 March 2024	10
15 min	1 March 2024	10

**Table 2:** Acquisition parameters.

Acquisition Parameters	Value
Excitation wavelength	280 nm
Emission wavelength	270 nm–600 nm
Excitation slit width	1.5 nm
Emission slit width	1.5 nm
Integration time	100 ms
Excitation scan step size	5.0 nm
Emission scan step size	1.0 nm

**Table 3:** Evaluation indicators of models after spectral preprocessing.

Evaluation Indicators	Original	SG	Median	Mean	Gaussian	Lowess	Loess	Rlowess	Rloess
MAE	1.8317	1.1962	1.3435	0.72386	0.80052	0.86717	0.97859	0.73532	1.1972
MSE	7.5201	2.1821	3.0601	1.2868	1.3843	1.5284	1.5227	0.94403	2.2853
RMSE	2.7423	1.4772	1.7493	1.1344	1.1766	1.2363	1.234	0.97161	1.5117
R^2^	0.70477	0.91433	0.87987	0.94948	0.94566	0.94	0.94022	0.96294	0.91028

**Table 4:** Evaluation indicators of models after dimensionality reduction.

Evaluation Indicators		Original	SG	Mean	Gaussian	Lowess	Loess	Rlowess
PCA	MAE	0.35217	0.31527	0.52965	0.70788	0.41558	0.53789	0.61502
	MSE	0.23524	0.14999	0.35175	0.83849	0.27578	0.49177	0.95938
	RMSE	0.48502	0.38728	0.59309	0.91569	0.52514	0.70126	0.97948
	R^2^	0.99076	0.99411	0.98619	0.96708	0.98917	0.98069	0.96234
KPCA	MAE	0.61273	0.86364	0.54661	0.62488	1.1149	0.68433	0.67522
	MSE	1.2029	1.2103	0.74745	0.56053	2.9142	0.64992	0.72844
	RMSE	1.0967	1.1001	0.86455	0.74868	1.7071	0.80618	0.85349
	R^2^	0.95278	0.95249	0.97066	0.97799	0.88559	0.97449	0.9714
MDS	MAE	0.60583	0.8366	0.43934	0.65861	0.5407	0.69559	0.43913
	MSE	0.57823	1.1271	0.35401	0.8975	0.54504	0.86949	0.34823
	RMSE	0.76042	1.0616	0.59499	0.94736	0.73827	0.93246	0.59011
	R^2^	0.9773	0.95575	0.9861	0.96477	0.9786	0.96587	0.98633

**Table 5:** Evaluation indicators of models after PSO optimized BPNN.

	Gaussian- PCA	Loess-PCA	Rlowess- PCA	Original-KPCA	Lowess- KPCA	SG- MDS	Mean- MDS
MAE	0.56972	0.46691	0.5522	0.73889	0.76997	0.32626	0.31647
MSE	0.57442	0.35049	0.54673	0.94695	0.84421	0.23481	0.20332
RMSE	0.7579	0.59202	0.73941	0.97311	0.91881	0.48457	0.45091
R^2^	0.97745	0.98624	0.97854	0.96282	0.96686	0.99078	0.99202

**Table 6:** Evaluation indicators of models after SBOA optimized BPNN.

	Rlowess- PCA	Original-KPCA	SG- KPCA	Gaussian-KPCA	Lowess- KPCA	Lowess- MDS	Loess- MDS	Rlowess- MDS
MAE	0.57971	0.42633	0.58447	0.30992	0.92728	0.51085	0.19345	0.33531
MSE	0.69486	0.53103	0.52652	0.22168	1.6783	0.46405	0.073678	0.25644
RMSE	0.83359	0.72872	0.72562	0.47083	1.2955	0.68121	0.27144	0.5064
R^2^	0.97272	0.97915	0.97933	0.9913	0.93411	0.98178	0.99711	0.98993

**Table 7:** Fluorescence spectral feature ratio value.

	0 min	2 min	4 min	6 min	10 min	15 min
FP	1.2782	1.3187	1.3290	1.3305	1.3569	1.3578

## Data Availability

The datasets used in this study can be accessed by contacting the corresponding authors with a valid request.
